# Identification of UL69 Gene and Protein in Cytomegalovirus-Transformed Human Mammary Epithelial Cells

**DOI:** 10.3389/fonc.2021.627866

**Published:** 2021-04-16

**Authors:** Sandy Haidar Ahmad, Fatima Al Moussawi, Ranim El Baba, Zeina Nehme, Sébastien Pasquereau, Amit Kumar, Chloé Molimard, Franck Monnien, Marie-Paule Algros, Racha Karaky, Thomas Stamminger, Mona Diab Assaf, Georges Herbein

**Affiliations:** ^1^ Department Pathogens & Inflammation-EPILAB EA4266, University of Bourgogne France-Comté, Besançon, France; ^2^ Molecular Cancer and Pharmaceutical Biology Laboratory, Lebanese University, Beyrouth, Lebanon; ^3^ Department of Pathology, CHRU Besançon, Besançon, France; ^4^ Institute for Clinical Virology, Ulm University, Ulm, Germany; ^5^ Department of Virology, CHRU Besancon, Besancon, France

**Keywords:** cytomegalovirus - HCMV, UL69, CTH cells, oncogenesis, latency

## Abstract

A growing body of evidence addressing the involvement of human cytomegalovirus (HCMV) in malignancies had directed attention to the oncomodulation paradigm. HCMV-DB infected human mammary epithelial cells (HMECs) in culture showed the emergence of clusters of rapidly proliferating, spheroid-shaped transformed cells named CTH (CMV-Transformed HMECs) cells. CTH cells assessment suggests a direct contribution of HCMV to oncogenesis, from key latent and lytic genes activating oncogenic pathways to fueling tumor evolution. We hypothesized that the presence of HCMV genome in CTH cells is of pivotal importance for determining its oncogenic potential. We previously reported the detection of a long non-coding (lnc) *RNA4.9* gene in CTH cells. Therefore, we assessed here the presence of *UL69* gene, located nearby and downstream of the *lncRNA4.9* gene, in CTH cells. The HCMV *UL69* gene in CTH cells was detected using polymerase chain reaction (PCR) and sequencing of *UL69* gene was performed using Sanger method. The corresponding amino acid sequence was then blasted against the *UL69* sequence derived from HCMV-DB genome using NCBI Protein BLAST tool. A 99% identity was present between the nucleotide sequence present in CTH cells and HCMV-DB genome. *UL69* transcript was detected in RNA extracts of CTH cells, using a reverse transcription polymerase chain reaction (RT-PCR) assay, and pUL69 protein was identified in CTH lysates using western blotting. Ganciclovir-treated CTH cells showed a decrease in *UL69* gene detection and cellular proliferation. In CTH cells, the knockdown of *UL69* with siRNA was assessed by RT-qPCR and western blot to reveal the impact of pUL69 on HCMV replication and CTH cell proliferation. Finally, *UL69* gene was detected in breast cancer biopsies. Our results indicate a close link between the *UL69* gene detected in the HCMV-DB isolate used to infect HMECs, and the *UL69* gene present in transformed CTH cells and tumor biopsies, further highlighting a direct role for HCMV in breast tumor development.

## Introduction

Human cytomegalovirus (HCMV) is a large double-stranded DNA virus of the Betaherpesvirinae subfamily, with a genome length of approximately 230 kbp (1). HCMV genome encodes for more than 700 short open reading frames (sORFs) and is composed of two unique regions, each flanked by inverted repeats (2, 3). During the lytic cycle, active viral genome replication results in the release of new viral particles, in contrast to the latency phase, during which a limited number of viral gene products are expressed. Latency is established depending on cell type-specified mechanisms related to transcriptional silencing unlike the reactivation phase triggered by diverse stimuli, for instance infection, inflammation, and injury (4). Studies have shown that HCMV activates pro-oncogenic pathways in infected cells, resulting in a modified cellular phenotype that favors transformation (5, 6). Indeed, HCMV gained a considerable attention in cancer due to many factors that could underlie its potential oncogenic role. The latter includes supporting proliferative signals, evading growth suppression while stimulating metastasis and invasion, permitting replicative immortality and angiogenesis, resisting cell death, escaping immune destruction, as well as inducing genome variability including mutations, and tumor stimulating inflammation (7, 8).

We previously observed that the infection with the clinical strain HCMV-DB resulted in the transformation of HMECs in culture, with the appearance of clusters of spheroid cells that contributed to the formation of CMV-transformed HMECs or CTH cells (6). When xenografted in NOD SCID gamma (NSG) mice, CTH cells underwent unchecked proliferation and gave rise to tumors (6). To determine the potential causal role of HCMV in the transformation of CTH cells, it is critical to detect the presence of HCMV genome in those transformed cells. Since the two oncogenic herpesviruses discovered so far, Epstein-Barr virus (EBV) and Kaposi’s sarcoma-associated herpesvirus (KSHV) exploit both latency and low lytic viral replication to fuel the oncogenic process (9–11), we decided to study HCMV genes and proteins expressed in CTH cells, even at low levels. In this context, studies have shown that few HCMV genes such as *UL123* and *UL122* immediate early genes, *UL7*, *UL111A*, *UL112*, *US28*, *US33*, *UL135*, *UL136*, and *lncRNA4.9* could be responsible for the tumorigenic phenotype observed in some human malignancies (12). Previously, we detected the presence of a 126 bp amplicon corresponding to the sequence of *lncRNA4.9* gene (nt95592- nt95717) of HCMV-DB strain in CTH cells (6). Since the previously detected *lncRNA4.9* is a non-coding sequence (6), we decided to further assess the presence of the nearby coding HCMV gene *UL69*, which might be a part of the transformation machinery.

Herein, we detected the presence of *UL69* gene in CTH cells using extensive PCR analysis and sequencing. Using phylogenic analysis, we confirmed that the detected *UL69* gene in CTH cells matches the *UL69* gene sequence of HCMV-DB strain originally used to infect and transform HMECs into CTH cells by 99%. At the proteomic level, the pUL69 sequence present in CTH cells differs from the HCMV-DB original pUL69 only in two amino acid point mutations. At the transcriptome level, we detected the transcript of *UL69* and pUL69 in CTH cells. Besides, in agreement with a coding role for *UL69* HCMV gene, we detected the expression of the pUL69 in CTH cells *in vitro*. Upon *UL69* knockdown using siRNA, we observed a decrease in viral replication and CTH proliferation. *In vivo*, we identified *UL69* gene with a high frequency in biopsies of breast cancer patients.

## Materials and Methods

### Isolation and Culture of CTH Cells

Several clusters of spheroid-cells were observed in HCMV-DB infected HMECs around day 20 post-infection in some of the cultures. These clusters were gently detached and the floating detached cells named CTH cells were cultured in HMEC Ready medium (Cat#12752010, Gibco, Grand Island, NY) for numerous passages, currently >150 passages (6). CTH cells used in this study were cultivated for approximately 410 days. HMECs cultures were infected with HCMV-DB at MOI of 1 as previously reported (13). An inverted light microscope (Olympus, Japan) was used to count cells in CTH cultures.

### Detection of the *UL69* Gene in CTH Cells Using PCR and qPCR Assay

Total DNA derived from uninfected HMECs, HCMV-DB infected HMECs, CTH, and MRC5 cells was extracted using a DNA extraction Kit (EZNA Blood DNA Kit, Omega BIO-TEK, Norcross, GA, USA). The presence of the HCMV sequence spanning the *UL69* gene was determined by qualitative PCR assay. The list of primer sets used to screen for the presence of *UL69* from HCMV-DB genome in CTH cells is described in **Supplementary Table 1**. Primer sets were provided by Eurogentec (Seraing, Belgium). Several Taq polymerases were used depending on the size and GC percent content of the amplified fragment. Dream Taq polymerase, KAPA hot start Taq polymerase, and KAPA fast Taq polymerase were ordered from Thermofisher (EP0701) and Sigma-Aldrich (KK1512 and KK4601) respectively. HCMV-DB DNA and lysates of HCMV-DB infected HMECs were used as positive controls. As negative controls, both uninfected HMECs and uninfected MRC5 cells were tested in parallel. Amplified products were electrophoresed in 2% agarose gel stained with Sybr green I nucleic acid stain. As an equal loading control, β-globin gene was amplified (sense: 5′-TCCCCTCCTACCCCTACTTTCTA-3′; antisense: 5′TGCCTGGACTAATC TGCAAGAG-3′). Using real-time qPCR assay, *UL69* load was measured in ganciclovir-treated (20 µM for 4 days) and untreated CTH cultures in parallel with the cell counting.

### Sequencing of the *UL69* Gene Present in CTH Cells

Fragments amplified by PCR were sequenced using the Sanger method (Genoscreen, Lille, France; GATC, Köln, Germany). PCR products were sent either directly after amplification or after gel cutting to harvest the band of the expected size (in case of several bands). **Supplementary Table 1** includes the primers used for sequencing. PCR amplifications and Sanger’s sequencing were repeated twice. The obtained sequences were prepared using the BIOEDIT software (14). Sequences were compared against the sequence of the HCMV-DB strain (accession number KT959235) (13) using NCBI nucleotide blast tool (https://blast.ncbi.nlm.nih.gov/BlastAlign.cgi).

### Detection of the *UL69* Transcript by RT-PCR

RNA was extracted from CTH cells using RNA extraction kit (EZNA Total RNA Kit I, Omega BIO-TEK). Following DNAse I treatment for 30 minutes (ThermoFisher), cDNA was synthesized from extracted RNA using Superscript IV First-strand synthesis kit (ThermoFisher) as per manufacturer instructions. cDNA was then used as a template for qualitative PCR amplification using *UL69* primers and Dream Taq polymerase (ThermoFisher) as described above. Lysates of HCMV-DB infected HMECs were used as positive controls. The amplified product was electrophoresed in 2% agarose gel stained with Sybr green I nucleic acid stain.

*UL69* knockdown with siRNA performed on CTH cells was assessed by the quantification of *UL69* RNA transcripts. Following RNA extraction and DNAse I treatment as described above, cDNA synthesis was performed. Real-time qPCR detection of UL69 was performed using KAPA SYBR FAST Master Mix (KAPA BIOSYSTEMS, KK4601) and *UL69* gene primers (sense: 5’-GGGATGTCGATGACTCCCTTC-3’; antisense: 5’- GTCGCTATTGGATCTCACCGT-3’), according to the manufacturers’ instructions. Real-time qPCR reactions were activated at 95°C for 10 minutes and then 50 cycles (15 s at 95°C and 1 min at 60°C) were conducted using a Stratagene Mx3005P thermocycler (Agilent Technology, Santa Clara, CA, USA). The results were recorded and analyzed using MxPro qPCR software.

### Western Blotting

Western blot was performed as described previously (13). Both controls and CTH cells were lysed and proteins were separated by electrophoresis on SDS-PAGE gel. Proteins were transferred to a nitrocellulose membrane. After blocking in 5% milk, the membrane was incubated overnight with 1 µg/ml anti-pUL69 polyclonal antibody (kindly provided by Dr. Stamminger, Erlangen University, Germany). Blots were developed with the ECL detection kit (Amersham Biosciences, Piscataway, NJ).

The siRNA-mediated knockdown of UL69 was assessed by western blot. Cellular extracts of transfected CTH cells with scramble siRNA and UL69 siRNA were prepared at day 1 post-transfection. In addition, cellular extracts of MRC5 infected with HCMV-DB and transfected with scramble siRNA and UL69 siRNA were prepared 24 hours post-transfection. β-actin was used as a control to normalize sample loading.

### Flow Cytometry Analysis

The proliferation of CTH cells was assessed using the measurement of Ki67 antigen expression by intracellular flow cytometry as described previously (15).

### Soft Agar Assay

Colony formation in soft agar seeded with ganciclovir-treated (20 µM for 4 days) and untreated CTH cells was assayed. Ganciclovir-treated and untreated CTH cells were incubated for 14 days in the semisolid agar medium. Colonies were observed under an Olympus microscope as previously reported (6).

### Transfection Assay

A total of 0.25×10^6^ cells (HMECs and CTH cells) were transfected with 1-2 µg total plasmid DNA, namely plasmids pUL69, empty pcDNA3.1 (16) and pGFP (17) using JetPEI transfection reagent (Polyplus Transfection, Illkirch, France) as per manufacturers’ protocol. pGFP-positive cells were gated and Ki67Ag expression was measured 24 hours post-transfection in pGFP-positive cells using flow cytometry.

### RNA Interference

HCMV-DB infected MRC5 and CTH cells were transfected with scramble siRNA or *UL69* siRNA (sense:ACUCAGCCGUUUGAUCGAATT; anti-sense:UUCGAUCAAAC GGCUGAGUTG) (Life Technologies, Carlsbad, CA, USA) using Lipofectamine RNAiMAX (Life Technologies) according to the manufacturer’s protocol. Transfection efficiency was monitored 24 hours post-transfection for HCMV-DB infected MRC5 cells by western blot and for CTH cells by RT-qPCR and western blot as described above.

### Determination of Amino Acid Sequences

Amino acid sequence derived from *UL69* gene present in CTH cells was obtained using the MBS translator software (http://insilico.ehu.es/translate/) (18). The *UL69* amino acid sequence existing in CTH cells was blasted against the pUL69 sequences derived from the HCMV-DB genome (KT959235) (13) using NCBI protein blast tool (https://blast.ncbi.nlm.nih.gov/Blast.cgi).

### Phylogenic Analysis

Multi-sequences alignments (MSA) were performed using CLUSTAL W. Phylogenic tree was obtained using the neighbor-joining method and MEGA7 software (http://www.megasoftware.net/) as previously described (6).

### Measurement of HCMV Growth

Viral replication was assessed by the quantification of HCMV load following DNA extraction (EZNA Blood DNA Kit, D3392-02, Omega BIO-TEK) by real-time qPCR detection using KAPA SYBR FAST Master Mix (KAPA BIOSYSTEMS, KK4601) and primers specific for the IE1 gene (sense: 5’-CGACGTTCCTGCAGACTATG-3’; antisense: 5’-TCCTCGGTCACTTGTTCAAA-3’) according to the manufacturer’s protocol. Real-time qPCR reactions were activated at 95°C for 10 minutes, followed by 50 cycles (15 seconds at 95°C and 1 minute at 60°C) using a Stratagene Mx3005P thermocycler (Agilent). Results were collected and analyzed using MxPro qPCR software.

### Detection of *UL69* DNA in Breast Tumor Tissue

All patients who were admitted to Besançon University Hospital (Besançon, France) gave their written informed consent to participate in the study according to the Helsinki declaration. The study was approved by the local ethics committees of Besançon University Hospital (Besançon, France) and the French Research Ministry (AC-2015-2496, CNIL n°1173545, NF-S-96900 n°F2015). Genomic DNA isolated from biopsies of breast cancer patients (n=30) was provided by the regional tumor bank (BB-0033-00024 Tumorothèque Régionale de Franche-Comté). Paired adjacent healthy tissue (n=29) was utilized in the study. 29 paired adjacent healthy samples match 29 tumor samples, but only one of the tumor samples lacks the corresponding healthy tissue. Breast tissues from non-tumor breast disease (n=4) were used as control. For histological grading of breast cancer biopsies, the Elston-Ellis grade was used with the three factors of grading: tubule formation, nuclear pleomorphism, and mitotic count. Each factor is given a score of 1 to 3 (1 being the best; 3 the worst) and the scores of all the three factors are then added up to give a total of 3 to 9 points indicating the tumor grade. Tumor grade is allocated on the following basis: 3 to 5 points: grade I (well-differentiated); 6 to 7 points: grade II (moderately differentiated); 8 to 9 points: grade III (poorly differentiated) (19). The presence of HCMV was determined by quantitative PCR using *UL69* primers (sense: 5’-GGGATGTCGATGACTCCCTTC-3’; antisense: 5’- GTCGCTATTGGATCTCACCGT-3’) as reported previously (6).

### Statistical Analysis

The reported values represent the mean and SD of independent experiments. Statistical analysis was performed using Mann Whitney U test and differences were considered significant at a value of *p*-value < 0.05. Microsoft Excel was used for plot construction. Correlation analysis was done using Spearman test (Statistical software SPSS 23). Based on Spearman’s rank correlation coefficient (rho), all bivariate correlation analyses express the strength of association between two variables where r = +1 (positive correlation) and the value r = -1 (negative correlation). Results were interpreted according to the degree of association as strong (0.7–1), moderate (0.5–0.7), or low (0.3–0.5), taking significant correlation (*p*-value < 0.05) values into consideration.

## Results

### Presence of HCMV-DB *UL69* Gene Signature in CTH Cells

**Figure 1:**
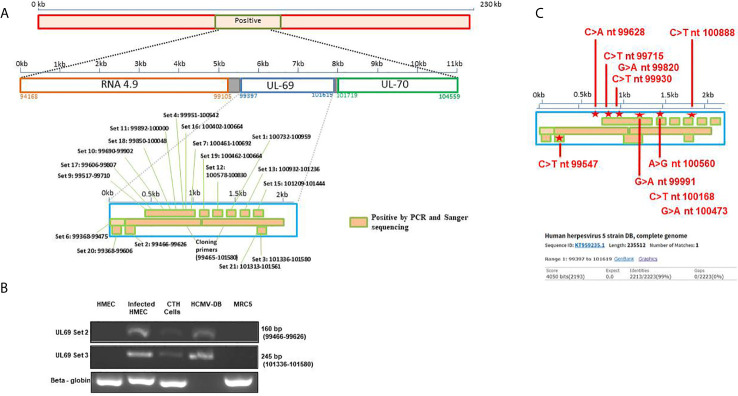
Detection of *UL69* gene in CTH cells. **(A)** Screening strategy used to detect HCMV *UL69* sequences in CTH cells using PCR assay. **(B)** *UL69* amplicons detection in CTH cells using conventional PCR. Uninfected HMECs and MRC5 cells were used as negative controls; HCMV-DB viral stock and infected HMECs were considered as positive controls. β-globin was used as an internal control. Agarose gels show results representative of three independent experiments. The complete primer sets used are described in **Supplementary Table 1**. **(C)** Detection of nucleotide point mutations in HCMV-DB *UL69* sequence present in CTH cells compared to wild-type HCMV-DB virus.

Since we previously identified *lncRNA4.9* sequences in CTH cells (7), we screened for the presence of the downstream and nearby *UL69* gene using primers spanning the complete gene region (**Figure 1A**, **Supplementary Table 1**). Using PCR assay, we first detected two HCMV-DB sequences in CTH cells corresponding to 160 bp and 245 bp *UL69* gene amplicons (**Figure 1B**), indicating that the complete *UL69* gene could be present in CTH cells. After demonstrating the presence of *UL69* genomic sequences in CTH cells, we used several primers sets to span the complete *UL69* gene (**Table 1**). Overall, we were able to detect the existence of a set of viral sequences in CTH cells that corresponds to the complete *UL69* gene (nt99397- nt101619) (**Figure 1B**, **Table 1**). *UL69* amplicons of the same size were also amplified in positive controls, including HMECs infected acutely with HCMV-DB and HCMV-DB viral stock (**Figure 1B**), whereas no HCMV sequences were detected in uninfected HMECs and uninfected MRC5 cells used as negative controls (**Figure 1B**).

**Table 1:** Amplicon positions and sizes of the *UL69* HCMV-DB gene screened in CTH cells using qualitative and/or quantitative PCR.

Gene	Amplicon position	Amplicon size (bp)	PCR amplification result in CTH cells
***UL69***	99368-99475	108	Positive
	99368-99606	258	Positive
	99465-101580	2115	Positive
	99466-99625	160	Positive
	99517-99710	194	Positive
	99606-99807	202	Positive
	99690-99902	213	Positive
	99850-100048	199	Positive
	99892-100000	109	Positive
	99951-100542	592	Positive
	100402-100664	263	Positive
	100461-100692	232	Positive
	100462-1000664	203	Positive
	100578-100830	253	Positive
	100732-100959	228	Positive
	100932-101236	305	Positive
	101209-101444	236	Positive
	101313-101561	249	Positive
	101336-101580	245	Positive

### Detection of Nucleotide Point Mutations in *UL69* Gene Present in CTH Cells Compared to the Original HCMV-DB Strain

To definitively confirm the presence of *UL69* gene in CTH cells, obtained *UL69* amplicons covering the complete gene were sequenced using the Sanger’s method. *UL69* sequences present in CTH cells were then compared to the HCMV-DB genome sequence (KT959235) by using BLAST analysis. We detected ten nucleotide point mutations in the *UL69* sequence obtained from CTH cells compared to the parental HCMV-DB sequence (**Figure 1C**, **Table 2**, **Supplementary Figure 1**). Thoroughly, a 99% identity was established between the *UL69* nucleotide sequence present in CTH cells and HCMV-DB genome (**Figure 1C**, **Supplementary Figure 1**).

**Table 2:** Detection of nucleotide point mutations in the *UL69* gene present in CTH cells compared to the HCMV-DB.

Mutation position	Nucleotide change between HCMV-DB and CTH cells	Codon change between HCMV-DB and CTH cells	Transcribed Codon change betweenHCMV-DB and CTH cells	Amino acid in HCMV-DB and CTH cells
**99547**	T → C	TTG→ CTG	CAA → CAG	Glutamine
**99628**	A→ C	ATC→CTC	GAU → GAG	Aspartic acid → Glutamic acid
**99715**	T→ C	TCG→CCG	CGA → CGG	Arginine
**99820**	A→ G	AGC→GGC	GCU → GCC	Alanine
**99930**	T→ C	TGT→TGC	ACA → GCU	Threonine → Alanine
**99991**	A→ G	ATG→GTG	CAU → CAC	Histidine
**100168**	T→ C	TGA→ CGA	UCA → UCG	Serine
**100473**	A→ G	CAA→CAG	UUG → CUG	Leucine
**100560**	G→ A	CAG→CAA	CUG → UUG	Leucine
**100888**	T→ C	TGA→CGA	UCA → UCG	Serine

### Detection of the *UL69* Transcript and Protein in CTH Cells

**Figure 2:**
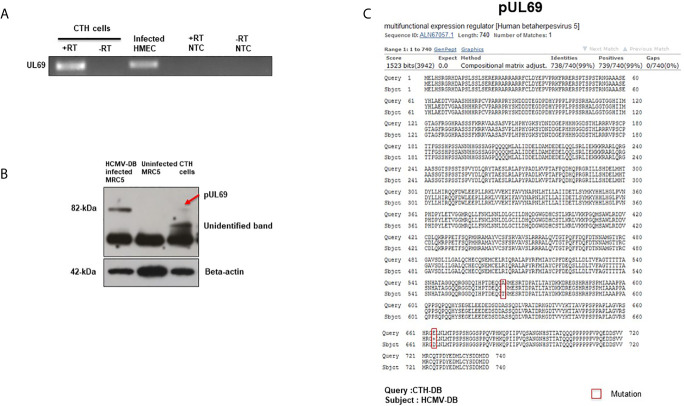
Detection of *UL69* transcript and pUL69 protein in CTH cells. **(A)** RT-PCR assay to detect the presence of *UL69* transcript in RNA extracts of CTH cells in the presence (+) and absence (-) of reverse transcriptase (RT). Infected HMECs were used as a positive control and no template control (NTC) as a negative control. **(B)** Detection of pUL69 protein in CTH cells by western blot. **(C)** Two residue point mutations present in pUL69 in CTH cells when compared to the original pUL69 from HCMV-DB isolate.

Following the detection of *UL69* sequence in CTH cells, we subsequently assessed the presence of *UL69* transcript and pUL69 protein in CTH cells. Using RT-PCR assay, we detected *UL69* transcript in RNA extracts of CTH cells (**Figure 2A**). We next analyzed the presence of pUL69 protein in CTH cells using western blotting. MRC5 cells infected with HCMV-DB were used as a positive control (**Figure 2B**). Despite its low levels, pUL69 protein was detected in CTH cells lysates at the expected size (82 kDa) (**Figure 2B**, red arrow). Intriguingly, one additional band of approximately 60 kDa was observed in CTH lysates compared to HCMV-infected MRC5 cells (**Figure 2B**, unidentified band). Amino acid sequence derived from *UL69* gene present in CTH cells was obtained using the MBS translator software (http://insilico.ehu.es/translate/) (18). UL69 amino acid sequence existing in CTH cells was blasted against pUL69 sequence derived from the HCMV-DB genome (KT959235) (13) using NCBI Protein BLAST tool (https://blast.ncbi.nlm.nih.gov/Blast.cgi). Two residue mutations were detected in pUL69 protein in CTH cells compared to the original HCMV-DB strain. The first mutation is at position 564, where the amino acid threonine (T) in HCMV-DB is changed to alanine (A) in CTH cells. The second one is at position 664, where the amino acid aspartic acid (D) is replaced by glutamic acid (E) (**Figure 2C**, **Table 2**).

### Phylogenetic Analysis of *UL69* Gene and pUL69 Protein Present in CTH Cells

**Figure 3:**
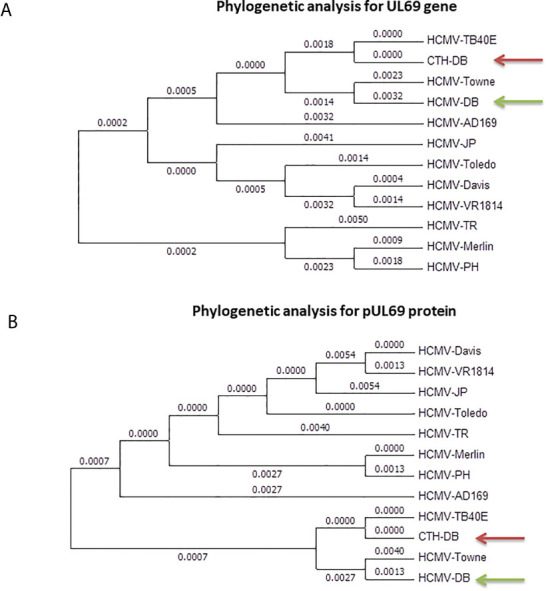
Phylogenetic analysis of *UL69* gene and pUL69 protein present in CTH cells. Phylogenetic analysis of *UL69* gene **(A)** and pUL69 protein **(B)** present in CTH cells compared to the original HCMV-DB strain in addition to other clinical and laboratory-adapted HCMV strains. This analysis was performed over three independent experiments.

*UL69* gene present in CTH cells was compared to its counterpart present in HCMV-DB genome, in parallel to comparison with ten other clinical and laboratory-adapted HCMV strains described previously (**Figure 3**) (5). We observed that *UL69* genomic sequence in CTH cells was mostly analogous to the *UL69* gene of TB40/E clinical strain than to that of the parental HCMV-DB strain (**Figure 3A**), indicating a shift of the *UL69* genomic sequence from HCMV-DB strain toward TB40/E strain. Similarly, at the protein level, we observed a shift from pUL69 protein of HCMV-DB strain toward TB40/E strain in CTH cells (**Figure 3B**). This analysis was performed over three independent experiments.

### Ganciclovir Treatment of CTH Cells Decreases Both *UL69* Gene Detection and Cell Proliferation

**Figure 4:**
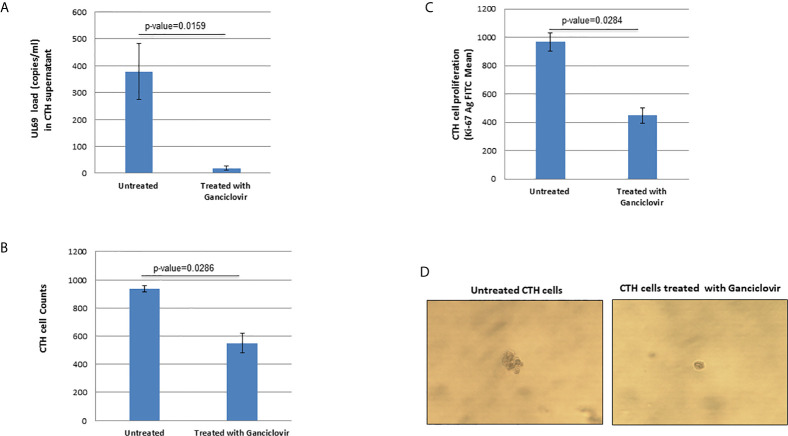
Ganciclovir treatment of CTH cells decreases *UL69* viral load, cell count, and cellular proliferation in culture and reduces colony formation in soft agar. **(A)** Untreated and ganciclovir-treated (20 µM for 4 days) CTH cells were compared for detection of *UL69* gene by qPCR, **(B)** cell counting under the microscope (total magnification of 200x), **(C)** cellular proliferation as measured by Ki67 antigen quantification by flow cytometry, and **(D)** colony formation in soft agar.

Since *UL69* and the previously described *lncRNA4.9* genes are detected in CTH cells, we assessed the presence of *UL69* gene in culture supernatants of CTH cells. We detected a very low viral load (around 2 log copies/ml) in culture supernatants of CTH cells, indicating the incidence of a relatively slow viral lytic cycle rate. Upon ganciclovir treatment, a 95% decrease in the viral load was detected in CTH cultures as measured by *UL69* quantification, further indicating that HCMV is replicating in CTH cells. Interestingly, parallel to the *UL69* decrease, ganciclovir treatment diminished the CTH cell count in culture by 41%, the CTH cell proliferation by 54%, and the size of the colonies observed in soft agar (**Figures 4A–D**).

### pUL69 Favors HCMV Replication and CTH Cell Proliferation

**Figure 5:**
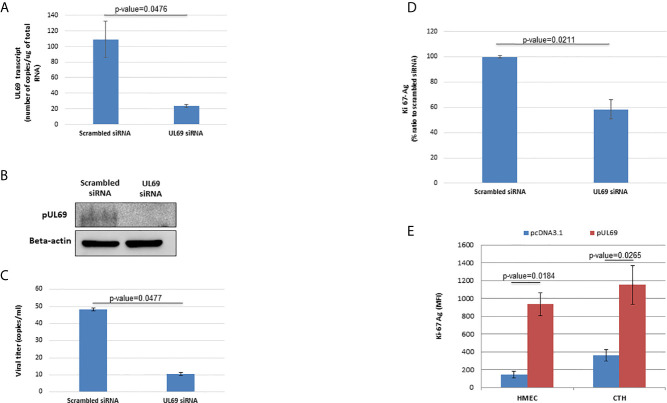
pUL69 favors HCMV replication and cellular proliferation in CTH cells. **(A, B)** Knockdown of *UL69* transcript and protein in CTH cells was monitored by RT-qPCR assay **(A)** and western blot **(B)** respectively. CTH cells were treated with *UL69* siRNA and a scramble control. RT-qPCR assay and western blot were performed as specified in Methods. **(C)** Knockdown of UL69 resulted in decreased HCMV replication in CTH cell cultures as measured by detection of IE1 in culture supernatants by qPCR assay. **(D)** *UL69* knockdown decreased CTH cell proliferation as measured by Ki67Ag quantification (MFI, mean fluorescence intensity) by flow cytometry. **(E)** Overexpression of pUL69 favors the proliferation of HMECs and CTH cells. Increased cellular proliferation as measured by Ki67 antigen quantification (MFI) by flow cytometry in HMEC and CTH cells transfected with pUL69 plasmid. Empty pcDNA3.1 transfected cells were used as control. Results are means (± SD) of three independent experiments.

To further clarify the role of UL69 protein in CTH cells, we treated CTH cells with *UL69* siRNA and a scramble control. Knockdown of *UL69* transcript and protein in CTH cells was monitored by RT-qPCR assay and western blot, respectively (**Figures 5A, B**). In addition, knockdown of UL69 protein was assessed in MRC5 cells acutely infected with HCMV-DB (**Supplementary Figure 2A**) in parallel with decreased HCMV replication in culture (**Supplementary Figure 2B**). Knockdown of *UL69* decreased both HCMV growth in CTH cell culture (**Figure 5C**) and CTH cell proliferation (**Figure 5D**). In addition, after overexpressing pUL69 in HMECs and CTH cells, we observed an increase in cell proliferation at 24 hours post-transfection using Ki67Ag detection by flow cytometry (**Figure 5E**). Altogether, our data indicate that *UL69* favors both HCMV replication and cell proliferation in CTH culture.

### Detection of *UL69* DNA Within Breast Cancer Biopsies

**Figure 6:**
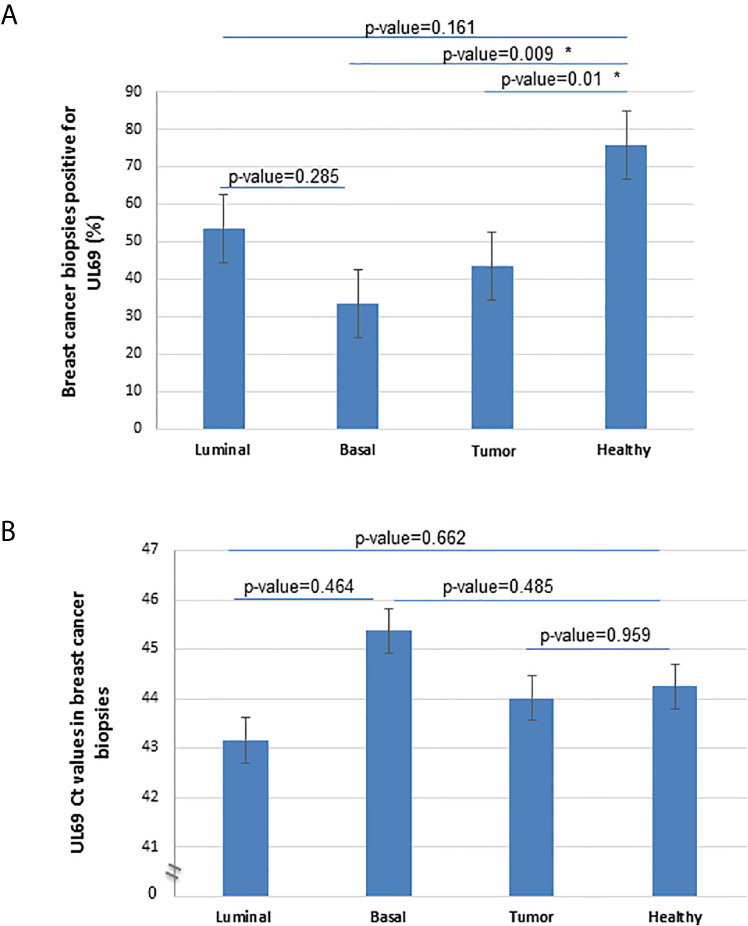
Detection of *UL69* DNA in human breast cancer biopsies. **(A)** Percentage of *UL69*-positive samples (Ct<50) in breast tumor biopsies (n=30), including luminal (n=15) and basal-like (n=15) tumors, and in adjacent healthy paired breast tissue (n=29) using *UL69* quantitative PCR measurement. **(B)** Quantification of HCMV *UL69* DNA in breast tumor biopsies (n=30) compared to healthy adjacent paired breast tissue (n=29), and in patients with basal-like breast cancer (n=15) compared to patients with luminal breast cancer (n=15), as measured by quantitative PCR *UL69* Ct value. * A statistically significant difference at *p*-value < 0.05. Histogram data are represented as mean ± SD of two independent experiments.

To further demonstrate the pathophysiological relevance of the CTH cell model *in vivo*, we assessed the expression of the *UL69* gene in the genomic DNA obtained from human breast cancer biopsies and paired adjacent healthy tissue. Breast cancer biopsies were classified into luminal and basal-like biopsies based on the IHC analysis of a pathologist. Breast tissue from patients with non-tumor breast disease was used as control. Using quantitative PCR, *UL69* gene was detected in 43.33% (n=13/30) and 75.85% (n=22/29) of biopsies from breast tumor and paired adjacent healthy breast tissue (**Figure 6A**) (*p*-value=0.01). In addition, *UL69* gene was detected in 53.33% (n=8/15) and 33.33% (n=5/15) of biopsies from luminal and basal-like breast cancer, respectively (**Figure 6A**). In breast tissue taken from patients with non-tumor breast disease, we detected the *UL69* gene in 50% of the cases in agreement with approximately 40%-50% of the French healthy adults harboring the virus (20) with a Ct value of 44.35. Among HCMV-positive (*UL69* gene positive) biopsies, we did not find significant differences in the viral load between paired healthy breast tissue (Ct=44.24), tumor breast tissue (Ct=44.01), luminal breast biopsies (Ct=43.16), and basal-like breast biopsies (Ct=45.38) as measured by quantitative PCR (*UL69* Ct value) (**Figure 6B**). In tumor biopsies, a significant positive correlation was detected between *UL69* Ct value and tubule formation (rho=0.53, *p*-value=0.038). In luminal breast cancer biopsies, a significant positive correlation was also found between *UL69* Ct value and tubule formation (rho=0.62, *p*-value=0.05). In addition, in basal-like breast cancer biopsies a significant negative correlation was observed between *UL69* Ct value and nuclear pleomorphism (rho=-0.89, *p*-value=0.05) (**Table 3**).

**Table 3:** Correlation between *UL69* Ct value and tumor grade (Elston-Ellis), tubule formation, nuclear pleomorphism and mitotic count in breast cancer biopsies.

	**Correlation between UL69 Ct value and**			
	Tumor grade	Tubule formation	Nuclear pleomorphism	Mitotic count
	***Rho***			
***All Tumor Biopsies***	0.22 (p>0.05)	0.53 (p=0.038)	0.12 (p>0.05)	-0.12 (p>0.05)
***Luminal Biopsies***	0.109 (p>0.05)	0.62 (p=0.05)	0.50 (p>0.05)	-0.29 (p>0.05)
Basal Biopsies*	NA	NA	-0.89 (p=0.05)	NA

*Since the values for Tumor grade, Tubule formation and Mitotic count in Basal biopsies are constant, the covariance of both variables is zero. Since a non-null covariance is required for correlation tests, the correlation cannot be applied to these constant values, making the correlation coefficient undefined.

## Discussion

Previously, we reported the transformation of HMECs infected with the HCMV-DB strain, namely the CTH cells (6). To substantiate the causative role of HCMV-DB in HMECs transformation, it is imperative to demonstrate the presence of coding HCMV-DB genes in CTH cells. One short DNA sequence related to the viral *lncRNA4.9* was previously detected in CTH cells. In the present study, we assessed the presence of *UL69* gene located downstream and nearby the *lncRNA4.9*. Upon sequencing the *UL69* gene present in CTH cells, we detected ten nucleotide point mutations that resulted in two amino acid point mutations in pUL69 in comparison with HCMV-DB genome. Remarkably, pUL69 protein, along with its transcript, were detected in CTH cells. Moreover, *UL69* gene was detected in breast cancer biopsies. Overall, our data denote the occurrence of the coding *UL69* gene in CTH cells, with potential pathophysiological relevance in breast cancer.

Earlier, we aimed to determine HCMV-DB signature in CTH cells by whole exome sequencing (WES) adopting Sureselect target enrichment method (21). Using WES, we detected limited sequences of HCMV-DB genomic DNA in the CTH cells genome, where the observed signals were close to the “background noise”. Among those weak WES signals in CTH cells, we noticed some hits corresponding to *lncRNA4.9* sequence of HCMV-DB. This was further confirmed by both qualitative and quantitative PCR that amplified a 126 bp fragment of *lncRNA4.9* originating from HCMV-DB, as confirmed by Sanger’s sequencing (6).

In the present study, *UL69* gene, its corresponding transcript, and pUL69 protein were detected in CTH cells confirming the availability of the *UL69* gene. The *UL69* gene covers a genomic region of 2.2 kb and encodes for 744 amino acids protein (pUL69) (22). pUL69 is a multifunctional regulatory protein belonging to the ICP27 herpesvirus family (23) which contains the ICP27 homology domain (IHD) (24). pUL69 could participate in the transformation of CTH cells *via* two distinct mechanisms: low viral replication levels in parallel to viral latency as already reported for the two oncoviruses (EBV and KSHV), and/or through a direct oncogenic potential of the viral protein (9–11, 25). Those two mechanisms might not be exclusive to each other. In agreement with low levels of viral replication in sustained CTH transformation by HCMV, we observed decreased levels of *UL69* gene in CTH cells treated with ganciclovir. In parallel, this was accompanied by a decrease in CTH cell count in culture, indicating reduced CTH cell proliferation, in addition to a lower number of colony formation in soft agar. Although we cannot exclude the role of other viral proteins in the maintenance of the CTH cell transformation throughout a prolonged cell culture, *UL69* gene and pUL69 might contribute to the exquisite balance between low viral replication and the transformation process. Thus, comparable to EBV and KSHV, HCMV low replication level could fuel oncogenesis, where pUL69 could be part of this process since it targets several stages of the lytic viral cycle such as transcription, mRNA nuclear export, and translation. Indeed, pUL69 is involved in gene transcription and binds *via* its IHD domain to the cellular transcriptional elongation factor SPT6 which favors chromatin remodeling. Upon knocking-down SPT6, an approximately 90% inhibition of the wild-type replication was detected, thus highlighting the significance of SPT6-UL69 binding in HCMV replication (24, 26, 27). Besides, pUL69 possesses binding motifs for UAP56/URH49 known as cellular mRNA adaptor proteins (28). pUL69 binding to the cellular helicase UAP56 has a role in transcriptional elongation and also promotes the mRNA nuclear export (16, 29). Additionally, pUL69 contains nuclear export and localization sequences that play a vital role in facilitating the shuttle between the nucleus and the cytoplasm (24). Finally, to modulate translation in HCMV-infected cells, pUL69 interacts with part of the cap-binding complex, eIF4A1, and poly (A) binding protein (30). The detection of low levels of pUL69 protein and low viral replication rate in CTH cells are in line with the previously described constrained viral replication in sustained long term cultures of cancerous cell lines infected acutely with HCMV (31, 32). In fact, it has been shown that persistent HCMV infection of tumor cells may lead to the selection of novel virus variants characterized by changes in the coding sequences for virus regulatory proteins that have lost their ability to induce cell cycle arrest. Thus, persistent HCMV infection of tumor cells results in the development of mutant viral variants, that grow slowly and yield lower amounts of progeny virus compared to the wild-type viral strains originally used for infection (31, 33).

Based on *UL69* knockdown (**Figures 5A–D**) and overexpression (**Figure 5E**) experiments, we observed that *UL69* favors both HCMV replication and cell proliferation in CTH cultures. Enhanced cell proliferation could be involved in the sustained growth of CTH cells in culture. In contrast to our data in CTH cells, a pUL69-mediated cell cycle block in G1/S phase has been reported previously in acutely HCMV-infected MRC5 cells (34, 35). The difference in the cell type used (fibroblast versus epithelial cells) and in acute (MRC5 cells) versus chronic (CTH cells) infection could explain the observed discrepancies. In addition, cells with disrupted cell cycle tend to control mechanisms in a similar manner to tumor cells; the function of virus regulatory proteins may depend on the internal landscape of tumor cells including p53 and Rb expression/mutation (36). For example, HCMV IE2 protein induces a G1/S block in human cells with wild-type pRb, but not in the human Rb-deficient osteosarcoma cell line Saos-2. Similarly, sustained HCMV infection did not induce cell cycle arrest in T89G glioblastoma cells with disrupted p53 signaling, but virus antigen-positive cells continued to divide (37). Interestingly, we previously reported both a functional defect of p53 and Rb downregulation in CTH cells infected with HCMV-DB (6). These findings indicate that the effects of HCMV on cell cycle and cell proliferation may depend on both the landscape of the internal cellular environment (e.g. p53, Rb) and the properties of virus regulatory proteins such as pUL69 (38). Although we did not yet decipher the molecular mechanism(s) involved in HCMV transformation and the role played by pUL69 (alone or in conjunction with other viral proteins) in this phenomenon, pUL69 favors sustained proliferation of CTH cells in parallel with low sustained viral growth in culture which have been reported as key elements for the transforming capabilities of the two oncogenic herpesviruses EBV and KSHV (25).

In addition to the role of pUL69 in low level viral replication detected in CTH cell cultures, it might also be directly involved in the oncogenic process fueled by HCMV. Notably, the functions of several cellular proteins involved in anti-viral responses and oncogenesis are under the control of certain modifications caused by the small ubiquitin-like modifiers (SUMOs), referred to as SUMOylation. These modifications can change the stability or localization of a specific protein and are capable of enhancing protein-protein interactions through the binding process of SUMOs to the SUMO-interacting sequences (SIMs) (39, 40). SUMOylation acts as a fundamental controller of cell cycle progression, genome stability, senescence, gene expression, stress, and innate immune responses (39, 41, 42). SUMO signal transduction has been identified as a key factor in the development of various cancer types, and SUMOylation was shown to be highly upregulated in several malignancies including breast cancer (39, 43–45). A recent report indicates that pUL69 has a SUMO E3 ligase activity and it can potentially bind to SUMO and Ubc9 (46). Besides, pUL69 takes over the induction of p53 SUMOylation when in complex with Ubc9 and SAE (46). Moreover, in Kaposi’s sarcoma-associated herpesvirus (KSHV), K-bZIP represents a SUMO2/3-specific E3 ligase, leading to its own SUMOylation in addition to that of p53 and Rb (47). Since pUL69 protein present in CTH cells possesses an E3 ligase activity, it might induce the inactivation of p53 through SUMOylation, thereby potentially leading to the development of cancer. In addition, a virally-encoded deSUMOylase activity is required for HCMV reactivation from latency (48). In line with the fact that SUMOylation is involved in HCMV latency, it is often considered as a prerequisite for cellular transformation by herpes oncoviruses (7, 25). It should be pointed out that the binding of pUL69 to PRMT6 could be another mechanism elucidating its role in cancer development. We previously noted the upregulation of PRMT6 gene expression in HCMV-infected HMECs (5). The binding of pUL69 to PRMT6 could favor oncogenesis as already reported with the upregulated expression of PRMT6 in prostate cancer (17, 49). Therefore, the multifunctional pUL69 protein might play a complex role in controlling CTH cell transformation. Future studies adopting *UL69* deletion and/or point mutation constructs and UL69-defective HCMV might further assess its direct (or indirect) role in oncogenesis.

It is worth noting that in macrophages, interferon (IFN) production is inhibited through ICP27‐mediated interaction with the active stimulator of interferon genes (STING) signalosome. Knowing that UL69 protein represents the HCMV homolog of ICP27, it compromises the STING signaling pathway and type I IFN production, which might favor viral immune evasion and further cancer cell proliferation and migration (50, 51). Furthermore, a previous study indicated a potential role of the viral immuno-modulator cytokine IL-10 during HCMV infection in enhancing breast tumor development and metastasis (52). Likewise, HCMV IL-10 exposed MCF-7 human breast cancer cells showed a significant up-regulation of matrix metalloproteinase-10 (MMP-10) gene expression, thus promoting excessive proliferation and tissue invasion (53).

Although *lncRNA4.9* has been shown to physically interact with the HCMV latent viral chromosome (54), we previously reported the presence of HCMV *lncRNA4.9* gene in breast cancer biopsies (6). In the present study, we detected the presence of the nearby *UL69* gene in breast cancer biopsies. A lower detection of the *UL69* gene (as measured by the percentage of *UL69* positive samples) was observed in breast tumor tissue compared to paired healthy breast tissue, in agreement with the previously reported block of HCMV replication in tumor tissue (55, 56). Since HCMV is detected in both tumor tissue and healthy tissue present in the vicinity of the breast tumor, the presence of HCMV in epithelial cells, tissue macrophages, or endothelial cells in normal breast tissue could fuel cellular transformation leading to breast cancer in some patients.

In conclusion, our findings highlight the potential of a clinical HCMV strain, namely the HCMV-DB isolate, to fulfill all the requirements contributing to the transformation of HMECs *in vitro*, causing the genesis of CTH cells. HCMV could possibly induce transformation through viral gene expression, including the *UL69* gene, which has been hijacked by latent and/or lytic viral cycle toward cellular transformation. The presence of *UL69* DNA in the majority of biopsies isolated from women with breast cancer indicates a potential relevance of *UL69* gene in breast cancer pathophysiology. The study of the *UL69* gene in CTH cells could be relevant to understand the molecular mechanisms involved in the development of breast cancer.

## Data Availability Statement

The datasets presented in this study can be found in online repositories. The names of the repository/repositories and accession number(s) can be found in the article/**Supplementary Material**.

## Ethics Statement

The studies involving human participants were reviewed and approved by the study was approved by the local ethics committees of Besançon University Hospital (Besançon, France) and the French Research Ministry (AC-2015-2496, CNIL n°1173545, NF-S-96900 n°F2015). Genomic DNA isolated from biopsies of breast cancer patients (n=30) was provided by the regional tumor bank (BB-0033-00024 Tumorothèque Régionale de Franche-Comté). The patients/participants provided their written informed consent to participate in this study.

## Author Contributions

SH, FAM, RB, ZN, SP, AK, FM and CM performed experiments. SP, GH, RK, M-PA, MD and TS participated to the data analysis. GH conceived and designed the project. SH, RB, SP and GH wrote the manuscript. All authors contributed to the article and approved the submitted version.

## Funding 

This work was supported by grants from the University of Franche-Comté (CR3300), and the Région Franche-Comté (2020-11890). The funders had no role in the data collection, analysis, patient recruitment, or decision to publish.

## Conflict of Interest

The authors declare that the research was conducted in the absence of any commercial or financial relationships that could be construed as a potential conflict of interest.

## Supplementary Material

Supplementary Figure 1 Blast analysis of *UL69* nucleotide sequence. Comparison of *UL69* gene sequence present in CTH cells (Query) to the wild type HCMV-DB (Subject).

Supplementary Figure 2 Assessment of pUL69 protein expression and HCMV growth in MRC5 cells treated with UL69 siRNA and siRNA scramble. **(A)** pUL69 expression and **(B)** HCMV growth were monitored 24 hours post transfection with siRNA in HCMV-DB infected MRC5 cells by western blot and detection of IE1 in culture supernatants by qPCR assay, respectively. Results are representative of two independent experiments.

## Abbreviations

A, adenine; C, cytosine; CDK19, cyclin dependent kinase 19; CTH cells, CMV Transformed HMECs; EBV, Epstein-Barr virus; G, guanine; HCMV, human cytomegalovirus; HMECs, human mammary epithelial cells; IFN, interferon; IHD, ICP27 homology domain; KSHV, Kaposi’s sarcoma-associated herpesvirus; LncRNA4.9, long non-coding RNA4.9; MMP-10, matrix metalloproteinase-10; MSA, multi-sequences alignments; NTC, no template control; PCR, polymerase chain reaction; RT, reverse transcriptase; RT-PCR, reverse transcription polymerase chain reaction; SIMs, SUMO-interacting sequences; sORFs, short open reading frames; STING, stimulator of interferon genes; SUMOs, small ubiquitin-like modifiers; T, thymine; WES, whole exome sequencing.
