# Supplementary material for: Identification of UL69 Gene and Protein in Cytomegalovirus-Transformed Human Mammary Epithelial Cells
Source: Front Oncol. 2021 Apr 16;11:627866. doi: 10.3389/fonc.2021.627866 (PMC8085531; doi:10.3389/fonc.2021.627866)
Supplement: Supplementary Figure 1 — Blast analysis of UL69 nucleotide sequence. Comparison of UL69 gene sequence present in CTH cells (Query) to the wild type HCMV-DB (Subject). [file DataSheet_1.pdf]

Supplementary Table 1 : Primer sequences and amplicon positions of the *UL69* HCMV-DB gene screened in CTH cells using qualitative and/or quantitative PCR.

Gene	Primers Sequence	Amplicon positions
UL69	FWD: 5' ATCATCACGCACAACACCCT 3'<br>RVS: 5' AGACGACTCGGTGGTAATGC 3'	99368-99475
	FWD: 5' ATCATCACGCACAACACCCT 3'<br>RVS: 5' TAACTTGATGACGCCGTCGC 3'	99368-99606
	FWD: 5' AGTCGGTACCCGAGTCGTCTTCTTGCGGTA 3'<br>RVS: 5' CGGACGTGAATTCTTATCTTCCCTGAGCGAGCG 3'	99465-101580
	FWD: 5' CGAGTCGTCTTCTTGCGGTA 3'<br>RVS: 5' TAACTTGATGACGCCGTCGC 3'	99466-99625
	FWD: 5' TGCCGTCGTACTGTGATTACC 3'<br>RVS: 5' GGGACACCGTCGTCTACAAG 3'	99517-99710
	FWD: 5' GCGACGGCGTCATCAAGTTA 3'<br>RVS: 5' CAACCACAGCAGCACTACTC 3'	99606-99807
	FWD: 5' TCTTGTAGACGACGGTGTCC 3'<br>RVS: 5' CAACGCTGACAGCCTACGA 3'	99690-99902
	FWD: 5' GGGATGTCGATGACTCCCTTC 3'<br>RVS: 5' GTCGCTATTGGATCTCACCGT 3'	99850-100048
	FWD: 5' TGTCAGCGTTGCTGGGTC 3'<br>RVS: 5' CAGCAACCATGCCACTGC 3'	99892-100000
	FWD: 5' GAATTTGGTCTCCTCCCCGC 3'<br>RVS: 5' AACCCGCACGATCCCTATCT 3'	99951-100542
	FWD: 5' GACTAACCGTTTGAGCGTGC 3'<br>RVS: 5' GCAAGCTGGTGGTGGAAAAG 3'	100402-100664
	FWD: 5' AGCCCAGGTCCAAGTTGTTG 3'<br>RVS: 5' TTCGATTGGCTGGAGGAACC 3'	100461-100692
	FWD: 5' GCCCAGGTCCAAGTTGTTGA 3'<br>RVS: 5' GCAAGCTGGTGGTGGAAAAG 3'	100462-100664
	FWD: 5' AGCTGAGCGTTTCGTCGAT 3'<br>RVS: 5' AGTCTACGTCTGGCACCCT 3'	100578-100830
	FWD: 5 CTCGTCGTGTGAACAGCAGGATG ' 3'<br>RVS: 5' GAACTACAGCAACTCAGCCGTTTGA 3'	100732-100959
	FWD: 5' CGATCAAACGGCTGAGTTGC 3'<br>RVS: 5' ACATAGGGCGTCCTCCTCTT 3'	100932-101236
	FWD: 5' TCGCTTGAAAGAGGAGGACG 3'<br>RVS: 5' GCGAATACCATCTCGCGGAA 3'	101209-101444
	FWD: 5' GGGGAGGAGGATAGTGGTCC 3'<br>RVS: 5' TTATCTTCCCTGAGCGAGCG 3'	101313-101561
	FWD: 5' TCGCCTTCGGTATCATCGTC 3'<br>RVS: 5' TTATCTTCCCTGAGCGAGCG 3'	101336-101580

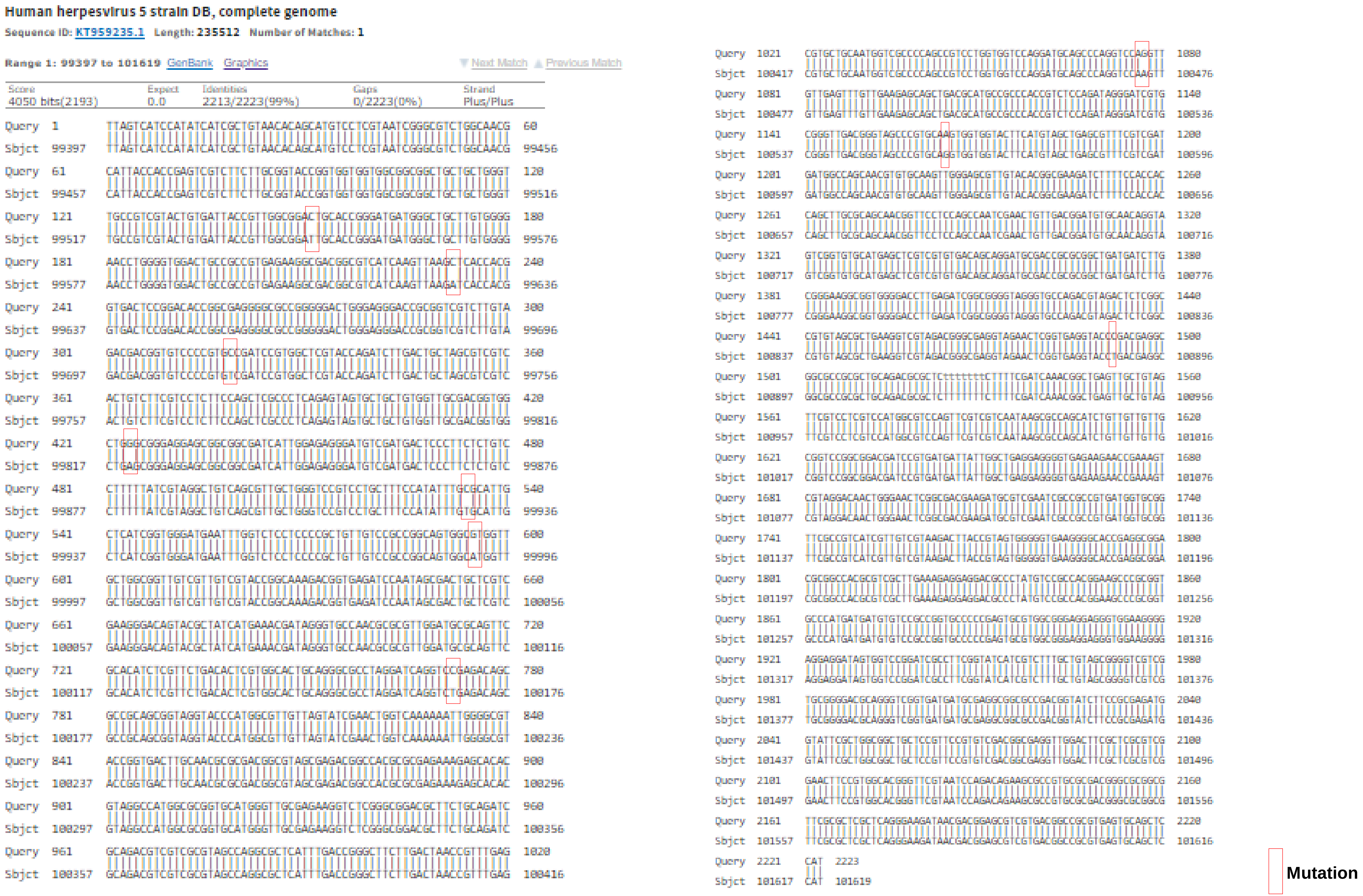
Human herpesvirus 5 strain DB, complete genome
Sequence ID: KT959235.1 Length: 235512 Number of Matches: 1
Range 1: 99397 to 101619 GenBank Graphics
Next Match Previous Match
Score 4050 bits(2193)
Expect 0.0
Identities 2213/2223(99%)
Gaps 0/2223(0%)
Strand Plus/Plus
Mutation

Sup. Figure 1

A

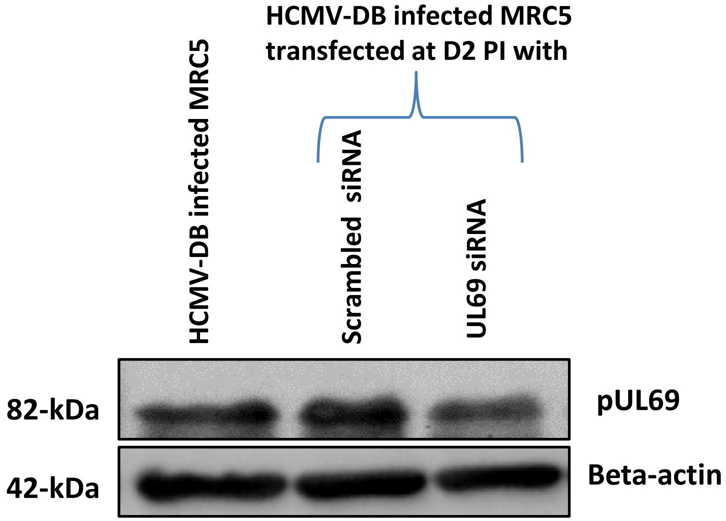

B

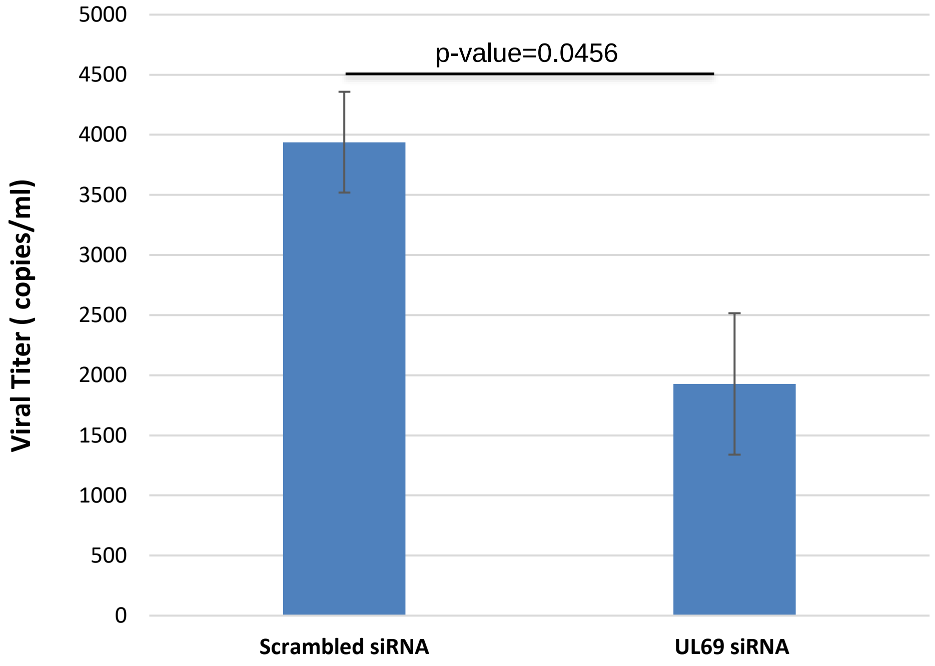

Sup. Figure 2
